# An Extra Cue Is Beneficial for Native Speakers but Can Be Disruptive for Second Language Learners: Integration of Prosody and Visual Context in Syntactic Ambiguity Resolution

**DOI:** 10.3389/fpsyg.2019.02835

**Published:** 2020-01-10

**Authors:** Chie Nakamura, Manabu Arai, Yuki Hirose, Suzanne Flynn

**Affiliations:** ^1^Global Center for Science and Engineering, Waseda University, Tokyo, Japan; ^2^Department of Linguistics, Massachusetts Institute of Technology, Cambridge, MA, United States; ^3^Faculty of Economics, Seijo University, Tokyo, Japan; ^4^Department of Language and Information Sciences, University of Tokyo, Tokyo, Japan

**Keywords:** contrastive prosody, referential ambiguity resolution, garden-path, eye-movements, second language processing

## Abstract

It has long been debated whether non-native speakers can process sentences in the same way as native speakers do or they suffer from certain qualitative deficit in their ability of language comprehension. The current study examined the influence of prosodic and visual information in processing sentences with a temporarily ambiguous prepositional phrase (“Put the cake on the plate in the basket”) with native English speakers and Japanese learners of English. Specifically, we investigated (1) whether native speakers assign different pragmatic functions to the same prosodic cues used in different contexts and (2) whether L2 learners can reach the correct analysis by integrating prosodic cues with syntax with reference to the visually presented contextual information. The results from native speakers showed that contrastive accents helped to resolve the referential ambiguity when a contrastive pair was present in visual scenes. However, without a contrastive pair in the visual scene, native speakers were slower to reach the correct analysis with the contrastive accent, which supports the view that the pragmatic function of intonation categories are highly context dependent. The results from L2 learners showed that visually presented context alone helped L2 learners to reach the correct analysis. However, L2 learners were unable to assign contrastive meaning to the prosodic cues when there were two potential referents in the visual scene. The results suggest that L2 learners are not capable of integrating multiple sources of information in an interactive manner during real-time language comprehension.

## Introduction

It is known that the human language comprehension system rapidly integrates both linguistic and non-linguistic information in forming sentence representations (e.g., Tanenhaus et al., 1995). Accumulating evidence suggests that acoustic characteristics of speech, known as prosody, are no exception. Many studies, for example, have shown that comprehenders immediately use prosodic cues to resolve ambiguities in sentence structure (e.g., Snedeker and Trueswell, 2003). Studies that investigated the immediate use of prosody have demonstrated that listeners adopt different sentence representations by quickly considering prosodic features and visually presented context information (Ito and Speer, 2008; Nakamura et al., 2012). These studies indicate that comprehenders can use and integrate multiple sources of information such as visual and auditory information to resolve structural ambiguities during real-time comprehension. However, it is unclear whether people who acquired another language later in their life, namely late second language learners, can process the ambiguities in the same manner as native speakers. The current study addresses this issue by examining the processing of ambiguous sentences, which require the integration of visual and prosodic information, with both native speakers and second language (L2) learners. Comparison of the results from the two populations can provide valuable insight into differences in the nature of their language competences. Specifically, we are interested in revealing whether the L2 processing is fundamentally different in some way from native speakers, or are L2 learners slower and less accurate compared to native speakers simply because their L2 competence is underdeveloped?

Previous research showed that language users analyze spoken language-specific information in order to build the correct syntactic structure. For example, Snedeker and Trueswell (2003) tested globally ambiguous structures such as “Tap the frog with the flower.” They found that participants adopted different interpretations between the two alternatively possible structures depending on the location of the prosodic boundary (see also Schafer et al., 1996; Speer et al., 1996; Kjelgaard and Speer, 1999; Snedeker and Casserly, 2010). Also, Ito and Speer (2008) provided evidence for the predictive use of contrastive accent during discourse comprehension. They showed that the presence of contrastive accent on the contrastive color adjective (e.g., “green” in “First, hand the blue ball. Next, hand the green ball.”) led listeners to anticipate the upcoming word. These studies demonstrated that listeners immediately use prosodic information in building sentence representations, supporting the view that language users integrate all available information, including prosody, in formulating a structural analysis and update the most likely analysis according to the input (see also Marslen-Wilson et al., 1992; Weber et al., 2006).

Relatively little is known about how prosodic cues influence the interpretation and comprehension in L2 processing. For example, several studies reported that L2 learners are, like native speakers, sensitive to the alignment of prosodic boundary and syntax (e.g., Harley et al., 1995; Schafer et al., 2000; Dekydtspotter et al., 2008; Nickels and Steinhauer, 2016). Other studies reported the difference between native speakers and L2 learners in the perception of intonational meaning (e.g., Pennington and Ellis, 2000; Akker and Cutler, 2003; Braun and Tagliapietra, 2011). These results are hard to be unified because they tested different types of prosody (e.g., prosodic boundary and pitch accents) and participants with different L1s at different proficiency levels. However, the results of these studies at least suggest that L2 learners are sensitive to certain types of prosodic features but their ability to use them in online comprehension appears to be limited in some situations. The current study aims to explore the source of such L2 learners’ potential limitations by examining how L2 learners can integrate prosodic information and visual context during sentence processing.

### Sentence Processing in L2

Past research that investigated the processing of language learners suggest that L2 learners also make use of different types of linguistic information to formulate sentence representations (e.g., Soares and Grosjean, 1984 for lexical information, Hahne, 2001; Weber-Fox et al., 2003 for semantic information, Weber-Fox and Neville, 1996; Hahne and Friederici, 2001; Frenck-Mestre, 2002; Sanders and Neville, 2003 for syntactic information), although there is a consensus that L2 processing is generally slower and less accurate compared to L1 processing (e.g., Cook, 1997; Frenck-Mestre and Pynte, 1997; Green, 1998). Recent studies have begun to look into exactly what accounts for the differences between L1 and L2 sentence processing. Particularly, studies that used the experimental measures that produce observations with high temporal resolution such as ERPs or eye-tracking during comprehension and that investigated the time-course of L2 processing demonstrated that one fundamental difference between L1 and L2 appears to be the timing, that is, when specific linguistic information is used during comprehension rather than whether it is used at all. For example, Martin et al. (2013) recorded ERPs while Spanish-L1 English-L2 participants read the sentence-final word that was either expected (1a) or unexpected (1b) in a given context.

(1a) She has a nice voice and always wanted to be a singer.
(1b) She has a nice voice and always wanted to be an artist.

Their results indicated that the increase of the N400 amplitude on reading the unexpected final nouns was smaller with L2 learners compared to native speakers. This does not suggest that L2 learners did not predict the sentence-final word because the results from an off-line cloze probability test showed they predicted the same words as native speakers. The results instead suggest that their prediction was somewhat *weaker* when compared to native speakers.

Similarly, Ito et al. (2018) investigated the predictions of phonological information in English by Japanese-L1 English-L2 participants using a visual-world paradigm. In their study, participants heard the sentences which ended with a highly predictable word such as in (2).

(2) The tourists expected rain when the sun went behind the …

Their results revealed that L2 speakers predicted the target word (cloud) and looked at the corresponding picture before hearing the word. However, such predictive eye-movements were delayed when compared to native speakers. Their results also showed that native speakers looked predictively at the phonologically related competitor object (clown) but L2 learners did not show compatible looks to the phonological competitor. Ito et al. (2018) argue that the delay in L2 learners’ predictive looks to the target object is due to L2 learners’ decreased cognitive capacity in L2 sentence processing. Since L2 learners are not as proficient as native speakers, their processing requires greater cognitive resources. Consequently, they are left with fewer resources for processing compared to native speakers, which are not enough for predicting both the target word and the phonologically related word. This argument is largely consistent with the “Reduced Ability to Generate Expectations (RAGE)” hypothesis (Grüter and Rohde, 2013), which suggests that L2 speakers have reduced ability to generate predictions during comprehension and this is the main cause for the differences between L1 processing and L2 processing.

### Processing Accounts in L2

Many previous studies have reported processing differences between native speakers and adult L2 learners. Based on the results, several different accounts have been proposed to explain how and why these differences occur. For example, the RAGE hypothesis proposed by Grüter and Rohde (2013) suggests that L2 learners are less capable of making predictions about upcoming information during comprehension compared to native speakers. Given that prediction plays an essential role in language processing and most sentences contain at least some form of local or global ambiguities (e.g., Altmann and Kamide, 1999; Kamide et al., 2003; Boland, 2005), this view predicts that L2 learners’ performance in integrating information to predict upcoming material is less efficient than native speakers in processing almost all types of sentences. Another model that has been proposed in the L2 literature is the Shallow Structure Hypothesis (SSH, Clahsen and Felser, 2006). The SSH suggests that L2 learner’s processing is fundamentally different from L1 processing because the two groups rely on different types of information. According to the SSH account, sentence representations that L2 learners compute during comprehension contain less syntactic details than those of native speakers. This account claims that L2 learners have general tendency to rely more on lexico-semantic information and rely less on syntactic information.

Sorace and her colleagues proposed another account called the Interface Hypothesis, according to which L2 learners experience difficulties specifically when the processing involves the integration of syntax with other domains of information external to the grammar, such as discourse information. Sorace and Serratrice (2009) showed that L2 learners experienced processing difficulty when discourse-pragmatic information needs to be integrated with grammatical knowledge. Their hypothesis predicts that L2 learners’ performance would differ from native speakers’ when they need to process sentences involving the integration of syntax and information of other domains. Specifically, the hypothesis suggests that processing is particularly problematic for L2 learners when it involves the interface between syntax and discourse or pragmatics, which are called *grammar-external* domains as their appropriateness depends on context. In contrast, L2 learners have less of a problem when processing involves the interface between syntax and semantics or morphology, which are called *grammar-internal* domains as they involve formal features and their appropriateness or grammaticality is rather categorical (Sorace and Serratrice, 2009; Sorace, 2011).

Importantly, the above accounts seem to have different implications about the performance of highly proficient L2 learners. For example, as most of the L2 studies showed with their behavioral data, the RAGE hypothesis assumes that the L2 parser is slower and less accurate than native speakers. The hypothesis does not necessarily leave out the possibility that L2 parsing performance would approximate to the native speaker level as their proficiency increases, resulting in little or no difference in processing difficulty between native speakers and highly proficient L2 learners. The view that the acquisition of native-like parsing is possible, at least for very advanced learners, is consistent with a continuity of parsing hypothesis and in fact some study assumes that native processing and L2 processing are fundamentally identical (Hopp, 2010). In contrast, the Interface Hypothesis assumes that the coordination of external interfaces is inherently problematic for L2 processing. Under this view, it is predicted that even highly proficient L2 learners would not possess the complete native-like grammars, and would experience processing difficulty when the processing requires the integration of different, grammar-external, sources of information.

### Integration of Information in L2 Processing

In evaluating these hypotheses, one study by Pozzan and Trueswell (2016) investigated whether L2 learners would have difficulty when different domains of information need to be integrated to resolve ambiguity of the sentence structure. Specifically, they examined whether L2 learners resolve temporary ambiguity in the sentence structure by using visually presented referential information, by comparing the processing of temporarily ambiguous sentences such as (3a) to an unambiguous counterpart such as (3b).

(3a) Put the frog on the napkin into the box.
(3b) Put the frog that’s on the napkin into the box.

Pozzan and Trueswell adopted the experimental design originally used in Tanenhaus et al. (1995), in which participants saw visual scenes, which either contained one referent (e.g., one frog on a napkin) or two referents (e.g., one frog on a napkin and the other on a book). If visually presented contextual information is used to disambiguate the temporarily ambiguous situation of the sentence structure, it is expected that the processing of (3a) would be easier when presented with a two-referent context than with a one-referent context. This is because the visually presented context makes the noun *frog* ambiguous between the frog that is on a napkin and the other frog that is on a book, and thus creates a situation where an additional modifier is necessary.

Pozzan and Trueswell (2016) revealed similar eye-movement patterns between L2 participants and native speakers, showing that both groups made more fixations to the incorrect destination object (e.g., an empty napkin) in the one-referent context than in the two-referent context on hearing the ambiguous PP. The only difference observed between the two groups was in the rate of incorrect responses for the act-out task, in which L2 learners made more errors than native speakers in moving the mentioned object to the correct destination in the one-referent context. This most likely reflects that L2 learners were less confident in rejecting the initially adopted interpretation compared to native speakers, indicating that the difference between native speakers’ and L2 learners’ processing appears to lie in L2 learners’ inability to revise initially-adopted interpretations.

Although Pozzan and Trueswell (2016) captured the important processing difference between native speakers and L2 learners in the late stage of structural revision (i.e., after the sentence ended), they observed no difference between the two groups in eye-movement patterns during the presentation of the sentence. One possible reason why native speakers’ and L2 learners’ difference was not evident while listening to the sentence is that the processing cost in Pozzan and Trueswell (2016)’s experiment setting was low given the visual information is highly noticeable and required less cognitive effort. Thus, it is still possible that the interfaces would pose a problem for L2 learners when the processing of multiple sources of requires higher cognitive effort. In other word, we might observe a significant difference between L1 and L2 processing in more resource-intensive situations that involve multiple interface. In addition, another concern for Pozzan and Trueswell (2016)’s study is that, their results may possibly be explained by L1 transfer. Given that Italian has the same word order as well as the same PP-attachment ambiguity as in English, Italian speakers may have transferred the L1 knowledge to process the structural ambiguity.

Thus, it would be desirable to test the speakers whose L1 does not share the same word order or the same PP-attachment ambiguity with English. In Japanese, all modifiers come before nouns in contrast to English in which prepositional phrases that modify nouns appear after nouns. Because of this word order, PP-attachment is strictly unambiguous in Japanese. Therefore, for Japanese-L1 speakers to resolve the PP-attachment ambiguity in English, they would need L2-spceific grammatical knowledge. This may predict certain processing differences between Japanese-L1 English-L2 learners and native speakers.

### Current Study

In order to address these issues, we tested the influence of contrastive pitch accent on referential ambiguity resolution with Japanese-L1 English-L2 learners. The previous studies that investigated the mapping between prosody and speakers’ intentions showed that pitch accents are associated with unique functional meanings (Liberman and Pierrehumbert, 1984; Beckman and Pierrehumbert, 1986; Pierrehumbert and Hirschberg, 1990). For example, in English, a pitch accent aligned with a word stress (annotated as H^∗^ in ToBI transcription, Beckman et al., 2004) is typically adopted in standard declarative utterances whereas L + H^∗^ accents are known to mark a contrast in a relevant discourse context (Beckman and Ayers Elam, 1997). The current study focuses on these two prosodic features (H^∗^ or L + H^∗^) and investigated how these types of pitch accents are processed within particular visual context by native speakers and L2 learners. Sentences with the prepositional phrase (PP) attachment ambiguity such as (4) were tested.^1^

(4a) Put the cake on the plate_H^*_ in the basket.
(4b) Put the cake on the PLATE_L+H^*_ in the basket.

The PP is ambiguous between a modifier for the preceding noun phrase (NP) or a modifier for the verb which functions as a destination. The ambiguous PP carried different accents which was either H^∗^ or L + H^∗^.

**FIGURE 1:**
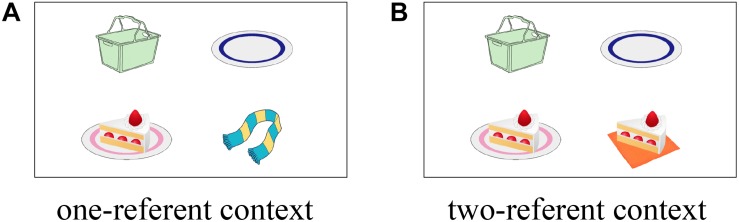
Example visual context for (4) in one-referent context condition **(A)** and in two-referent context condition **(B)**.

To manipulate discourse context, we used a visual scene that contained either one or two potential referents (Figure 1), following Tanenhaus et al. (1995). The visual scenes contained four entities, and three of them were the referents in the scene; the first-mentioned noun (cake), the second-mentioned noun in the ambiguous PP (empty plate as an incorrect destination), and a third-mentioned noun in the disambiguating PP (basket as a correct destination). The fourth object was manipulated to create two contextual conditions. In the one-referent condition, the fourth object was something that is not related to the sentence, which served as a distractor (a scarf in Figure 1A; one-referent context). In the other condition, the fourth object created a contrast to the potential referent (i.e., another cake on a napkin as opposed to “the cake on the plate” in Figure 1B; two-referent context).

Tanenhaus et al. (1995) who originally devised this manipulation was criticized for having a greater number of relevant pictures in the two-referent context condition might have eliminated the looks to the incorrect destination object, irrespective of how listeners analyzed the ambiguous PP. This possibility was explored and ruled out by Spivey et al. (2002), who included the “thee-and-one referent” context condition in their manipulation. In this condition, the competitor referent in the two-referent context (e.g., the other cake on a napkin) was replaced with a group of three cakes. Although there were two images that can be referred to by the noun “cake,” only the single cake was uniquely identifiable by the reference with a definite article “the cake” as listeners would not know which cake among the three is being referred to in this condition. Thus, if the looks to the incorrect destination object were eliminated by the presence of two potential referents in the two-referent context, the incorrect destination object in the three-and-one referent context should attract more fixations compared to that in the two-referent context. Spivey et al. (2002)’s results revealed no difference in the looks to the incorrect destination object between the three-and-one referent context condition and the two-referent context condition, providing evidence that the proportion of looks to the incorrect destination object is in fact reflecting the effect of a temporary misanalysis. Based on the results of Spivey et al. (2002), we consider that the incorrect analysis of attaching the ambiguous PP to the verb would be reflected in fixations to the incorrect destination object for the duration of the ambiguous PP. Consequently, how quickly participants reached the correct interpretation would be reflected in fixations to the correct destination object for the duration of the disambiguating PP.

Crucially, only the two-referent context involves referential ambiguity and therefore the contrastive pitch accent is informative for listeners only in this context but not in the one-referent context, which does not include a contrastive set. If comprehenders are sensitive to the contextual information and can immediately use the information to disambiguate the sentence structure, we predict that the contrastive pitch accent would help listeners to achieve the correct structural analysis. Our question is whether native speakers and L2 learners would show this pattern in the same manner or show a different pattern of results. Based on the previous studies, we have several reasons to believe that the latter possibility is likely. Below, we outline the detailed predictions for the patterns of results between native speakers and L2 learners.

Native speakers should interpret the L + H^∗^ pitch accent on the ambiguous PP as contrastive in presence of referential ambiguity. Previous studies indicate that L + H^∗^ contrastive pitch accents are used to signal a contrast discourse context, and listeners use the signal in identifying an upcoming referent in the processing of information structure (Ito and Speer, 2008; Ito et al., 2012). Thus, it is predicted for native speakers that L + H^∗^ pitch accent on the ambiguous PP in (4b) would highlight a contrast between the two cakes when presented with the two-referent context (Figure 1B) and thereby facilitate the correct Modifier PP analysis.^2^ On the other hand, such a facilitatory effect is not expected to occur when the L + H^∗^ accent is used in the one-referent context (Figure 1A). Previous research in speech production suggests that pragmatic functions of different pitch accents such as H^∗^ and L + H^∗^are not categorical but rather continuous (Ladd and Morton, 1997; Grice et al., 2017). This raises the possibility that comprehension of the pragmatic functions of pitch accent are highly context-dependent and that the identical pitch accent could convey different pragmatic intentions when used under different contexts. Assuming this is the case, the L + H^∗^ accent on the ambiguous PP in the one-referent context would be interpreted as simple prominence and encourage the destination interpretation. Hence, the contrastive accent in the one-referent context may boost the likelihood of the incorrect Destination PP analysis and cause a strong garden-path effect.

For L2 learners, on the other hand, this highly complex processes may be difficult and they may exhibit a different pattern of results. One possibility is that L2 learners cannot altogether process prosodic as well as visual information in the current study. This is the prediction based on the Interface Hypothesis, according to which L2 learners tend to have processing difficulties in external interface domains. This predicts a difference neither between the two types of prosody nor between the two types of referential contexts. However, as mentioned earlier, there is some evidence that L2 learners can process information in external interface domains (i.e., syntax and visually-presented contextual information, Pozzan and Trueswell, 2016), suggesting that L2 learners can process information in an external interface domain if it is highly salient and demands relatively little cognitive efforts. This suggests another possibility that L2 learners may be able to process visually presented contextual information but not prosodic information which is thought to be rather subtle for L2 learners. Specifically, if L2 learners cannot integrate the contrastive meaning of prosody with contextual information, it is possible that L2 learners may interpret the L + H^∗^ accent as emphatic regardless of the type of visual context. If this is the case, the contrastive accent, which is helpful for native speakers to process structural ambiguity in the two-referent context, may lead L2 learners to an incorrect parse.

## Experiment

### Experiment 1: Native Speakers of English

#### Participants

Twenty native speakers of English with normal visual acuity and hearing participated in the experiment. They were recruited in the Boston area, and received monetary compensation for participation.

#### Ethics Statement

This study was conducted with the approval of the Massachusetts Institute of Technology Committee on the Use of Humans as Experimental Subjects. Informed consent was obtained both verbally and in a written form from all participants.

#### Stimuli

**FIGURE 2:**
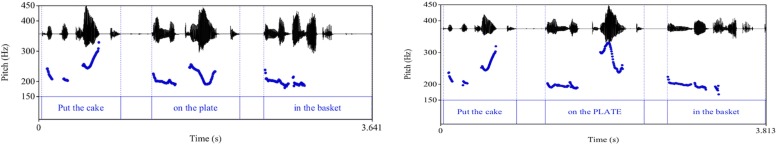
Waveform, pitch track, and accent type of sentence with H^∗^ **(left)** and L + H^∗^ **(right)**.

Twenty-four experimental items were created. For each item, we prepared an auditory sentence and a corresponding visual scene. The speaker for the auditory stimuli was a female native speaker of American English. Figure 2 shows the F0 contours of the sentence (4) with either H^∗^ or L + H^∗^, which associated with new information accent and contrastive accent respectively. The sentences in each item between the conditions were identical up to the onset of the ambiguous PP. In the post-focal disambiguating PP region, there was no difference in F0 peaks between H^∗^ and L + H^∗^ conditions.

We prepared the visual scenes using commercial clipart images. The position of objects in the visual scenes was counter-balanced across items. In addition to the 24 target items, 48 filler items were included. Four experimental lists were created using the Latin square design, and each list was presented in a pseudo-random order.

#### Procedure

Participants received brief instructions before they started the experiment. They were asked to listen to the sentences played from the audio speakers, and pay attention to the picture that was simultaneously shown on the computer screen. After each sentence was played, participants saw a mouse cursor. They were asked to click on the destination object for the fist-mentioned object in the sentence [i.e., the basket in Figure 1 with sentence (4)]. With this response, we can examine whether the ambiguous PP was correctly interpreted as a modifier for the preceding noun, or incorrectly interpreted as a destination. The moment-to-moment changes in eye-movements can tell us how this interpretation took place as they listened to the auditory input. In each trial, the auditory sentence was played 3000 ms after the picture was shown on the computer monitor. We recorded participants’ eye-movements using EyeLink 1000 (SR Research) at a sampling rate of 1000 Hz. Participants underwent a calibration procedure before the experiment started. The experimental session took approximately 30 min in total.

### Experiment 2: Japanese L2 Learners of English

#### Participants

Twenty-Nine Japanese learners of English, recruited from The University of Tokyo, Japan, participated in Experiment 2. All of the L2 participants had at least 6 years of English education before they enrolled in the university. We obtained participants’ score for the standardized English test in the National Center Test for University Admissions. Our L2 participants’ score corresponded to the proficiency level of C (Proficient) on the Common European Framework of Reference for Languages (CEFR).

#### Ethics Statement

This study was conducted with the approval of The University of Tokyo research subject review board. Informed consent was obtained both verbally and in a written form from all participants.

#### Stimuli and Procedure

Stimuli and procedure were identical to those in Experiment 1. In Experiment 2, we used Tobii TX300 to record participants’ eye-movements.

### Data Analysis and Results

The mean accuracy for the mouse click task was 99.51% for the native speaker’s group and 96.1% for the L2 learner’s group. The gaze location to the objects in the visual scene was converted from the fixation coordinates from the eye-tracker, which were mapped onto the four objects and the background. The onsets of the ambiguous PP (“on the plate”) and the disambiguating PP (“in the basket”) in each target sentence were marked manually. For the analysis, we first summed the looks to each object in the visual scene. We then calculated the logit of looks to each object out of looks to all the objects in the scene, including the background (Baar, 2008). We conducted statistical analyses for the duration of the ambiguous PP and the disambiguating PP using linear mixed effects regression models (Baayen et al., 2008). The dependent variable in the analyses is the logit of looks to the target object (incorrect destination or correct destination) out of looks to all the objects in the scene including background. The logit of looks to the target object is averaged over the time window of analysis per trial, time-locked to the onset of either the ambiguous PP or the disambiguating PP for each item. Since the analysis is time-locked to the onset of auditory presentation of the target word for each item, it does not discard timing information. Analyzing eye-movement patterns between conditions in the specified time windows allows us to see the difference between the conditions after participants received particular linguistic input and before they heard next input (such as from the onset of ambiguous PP up to the onset of disambiguating PP).

In all analyses reported here, we used linear mixed effect models in RStudio version 1.1.453 (RStudio Team, 2015), and packages lme4 version 1.1.19 (Bates et al., 2015) and lmerTest version 2.0.32 (Kuznetsova et al., 2017). In the model, Prosody (H^∗^ or L + H^∗^) and Visual Context (one-referent context or two-referent context), as well as the interaction between the two were included as fixed effects. Participants and items were included as random factors. We explored the best-fit model with an optimal random slope structure by using a backward selection approach. β, *t*-values, Standard Errors, and *p*-values from the optimal model are reported for each analysis. For the further analysis where a significant interaction was found, we applied dummy coding (0 and 1) for one fixed factor while keeping the coding for the other the same (i.e., effect-coding, −0.5 and 0.5) in the optimal model. For example, to examine simple effects of Prosody, we used the dummy coding for Visual Context, setting the reference level (0) for the level of Visual Context in which a simple effect of Prosody is examined. This allows us to test simple effects of Prosody without limiting the analysis to a subset of the data and causing α-level inflation due to multiple comparisons (the coefficient of the interaction remains constant, Aiken and West, 1991). Below, we first directly compare the results of native speakers and L2 learners in looks made to the correct destination object for the duration of the disambiguating PP. We then report the results of native speakers and L2 learners separately.

#### Combined Analysis Between Native Speakers and L2 Learners

First, we conducted a combined analysis on the looks made to the correct destination object (i.e., the basket in Figure 1) for the duration of the disambiguating PP (“in the basket”). The analysis was conducted for the 1000 ms time interval, 200 to 1200 ms following the onset of the disambiguating PP. In the model, Group (L1 and L2) was added as an additional categorical between-subject variable. In addition, the three-way interaction between Prosody, Visual Context, and Group was added in the model. This combined analysis conducted on the looks to the correct destination object showed there was a significant three-way interaction of the three factors (β = −2.79, *SE* = 0.89, *t* = −3.14, *p* = 0.002). The three-way interaction indicates that the pattern of the interaction between Prosody and Visual Context was significantly different between native speakers and L2 learners.^3^

In order to closely explore the different patterns in eye-movements between native speakers and L2 learners, we next analyzed native speakers and L2 learners separately for the looks made to the incorrect destination object (e.g., the empty plate in Figure 1) for the duration of ambiguous PP (“on the plate”) and those made to the correct destination object for the duration of disambiguating PP.

#### Separate Analyses: Native Speakers

**FIGURE 3:**
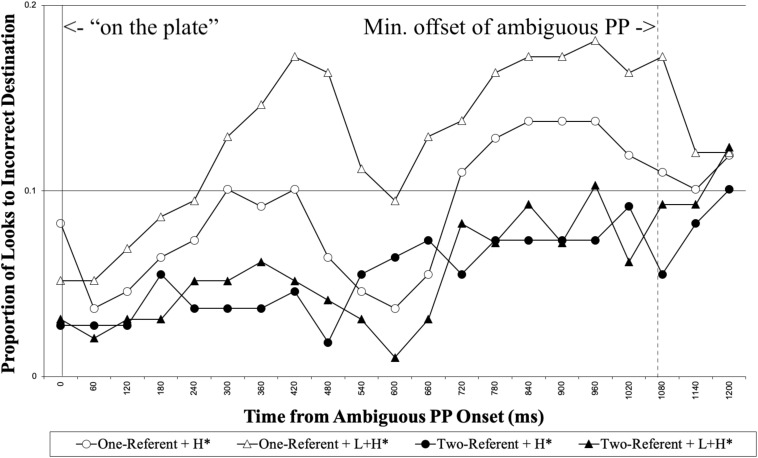
Native speakers’ proportion of looks to the incorrect destination object for the duration of the ambiguous PP, time-locked to the onset of the ambiguous PP (0 on the x-axis). The right vertical line marks the minimum offset of the ambiguous PP.

We first report the looks made to the incorrect destination object (e.g., the empty plate in Figure 1) for the duration of ambiguous PP (“on the plate”). Figure 3 shows the proportion of looks to the incorrect destination object for each 60 ms, time-locked to the onset of the ambiguous PP until 1200 ms following it.

We analyzed the looks to the incorrect destination object during the interval between the ambiguous PP onset to the minimum onset of the disambiguating PP (1057 ms). The first 200 ms was excluded from the analysis, considering the time required for linguistic information to influence eye-movements. Table 1 summarizes the results from the optimal model. The analysis showed there was a main effect of Visual Context, demonstrating that participants made more looks to the incorrect destination object in the one-referent context than in the two-referent context. The results indicate that native speakers adopted the incorrect Destination PP analysis more in the one-referent context than in the two-referent context on hearing the ambiguous PP. This finding is precisely what we expected to find, and is consistent with Tanenhaus et al. (1995), suggesting that visual context helped the listeners to interpret the ambiguous PP correctly. There was, however, no effect of Prosody in this time window.^4^

**TABLE 1:** Results from the model on looks to the incorrect destination object for the duration of the ambiguous PP.

	**β**	***SE***	***t***	***p***
Intercept	–5.45	0.20	–27.88	<0.001
Prosody	–0.44	0.31	–1.44	0.150
Visual context	–1.21	0.31	–3.93	<0.001
Prosody^∗^Visual Context	1.04	0.62	1.68	0.094

**FIGURE 4:**
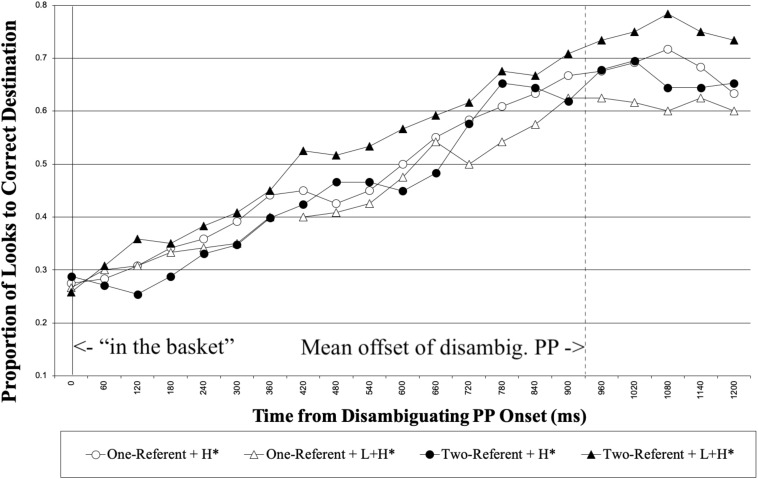
Native speakers’ proportion of looks to the correct destination object from the onset of disambiguating PP. The right vertical line marks the mean offset of the disambiguating PP.

Next, we report the analysis on the looks made to the correct destination object (i.e., the basket in Figure 1) for the duration of the disambiguating PP (“in the basket”). Figure 4 shows the proportion of looks made to the correct destination object for each 60 ms, time-locked to the disambiguating PP onset until 1200 ms following it.

We analyzed the looks made to the correct destination object for the 1000 ms time interval, 200 to 1200 ms following the onset of the disambiguating PP. Table 2 summarizes the results from the optimal model. No main effect was observed in the analysis. We observed an interaction between Prosody and Visual Context. Separate analyses on the simple effect of Prosody for the two context conditions revealed that the pattern of the effect of Prosody was opposite between the two context conditions. In the two-referent context condition, participants looked more at the correct destination object with L + H^∗^ than with H^∗^ (β = 0.99, *SE* = 0.46, *t* = 2.15, *p* = 0.032). In the one-referent context condition, participants looked more at the correct destination object with H^∗^ than with L + H^∗^, although the effect of Prosody did not reach the significance level (β = −0.61, *SE* = 0.46, *t* = −1.34, *p* = 0.154). Consistent with the predictions, the results demonstrate that the contrastive accent on the ambiguous PP facilitated the correct Modifier PP analysis when the visual scene contained a contrastive object set in the two-referent condition.^5,6^

**TABLE 2:** Results from the model on looks to the correct destination object for the duration of the disambiguating PP.

	**β**	***SE***	***t***	***p***
Intercept	1.54	0.36	4.33	<0.001
Prosody	0.19	0.32	0.57	0.566
Visual context	0.48	0.33	1.48	0.139
Prosody^∗^Visual Context	1.60	0.65	2.45	0.015

In the one-referent condition, it was suggested form the direction of the interaction that native speakers tended to be slower to reach the correct destination analysis with the contrastive accent on the ambiguous PP, possibly reflecting that native speakers tried to reconcile the incoherent use of contrastive accent without a contrastive set in the context, and as a result experienced processing difficulty.

#### Separate Analyses: L2 Learners

Analyses on looks to each object were performed in the same manner for the same intervals as in the analyses on native speaker’s data. In order to explore the relationship between L2 learners’ eye movement patterns and their English proficiency level, we also calculated z-score of each participant’s English test score and entered the score as an additional factor (Proficiency) in the LME model.

**FIGURE 5:**
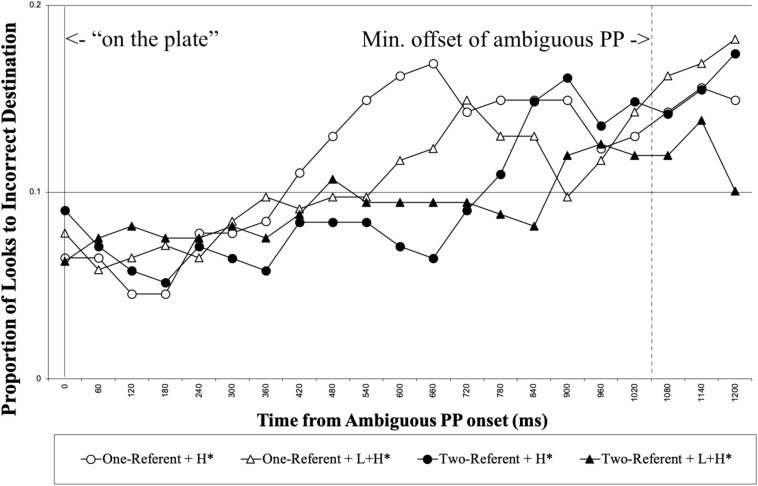
L2 learners’ proportion of looks to the incorrect destination object for the duration of the ambiguous PP, time locked for the onset of the ambiguous PP (0 on the x axis). The right vertical line marks the minimum offset of the ambiguous PP.

We first report the looks made to the incorrect destination object for the duration of the ambiguous PP. Figure 5 shows the proportion of looks to the incorrect destination object for each 60 ms, time-locked to the onset of the disambiguating PP. As in Experiment 1, the analysis was conducted in the time interval of 200 to 1257 ms, from the onset of the ambiguous PP until the minimum onset of the disambiguating PP. Table 3 summarizes the results from the optimal model.

**TABLE 3:** Results from the model on looks to the incorrect destination object for the duration of the ambiguous PP.

	**β**	***SE***	***t***	***p***
Intercept	–3.89	0.29	–12.99	<0.001
Prosody	–0.01	0.27	0.02	0.985
Visual context	0.58	0.27	2.20	0.028
Proficiency	–0.47	0.27	–1.77	0.088
Prosody^∗^Visual Context	–0.71	0.54	1.32	0.189

The analysis showed there was a main effect of Visual Context; participants looked at the incorrect destination object more in the one-referent context condition than in the two-referent context condition. The results suggest that L2 learners adopted the correct interpretation more in the two-referent condition than in the one-referent condition on hearing the ambiguous PP. The results are consistent with the native speaker’s results, demonstrating that L2 learners adopted the correct Modifier PP analysis more when the visual context had a contrastive object set. The results provide evidence that referential visual context information helped L2 learners to resolve the temporarily ambiguous structure of the sentence.^7^

**FIGURE 6:**
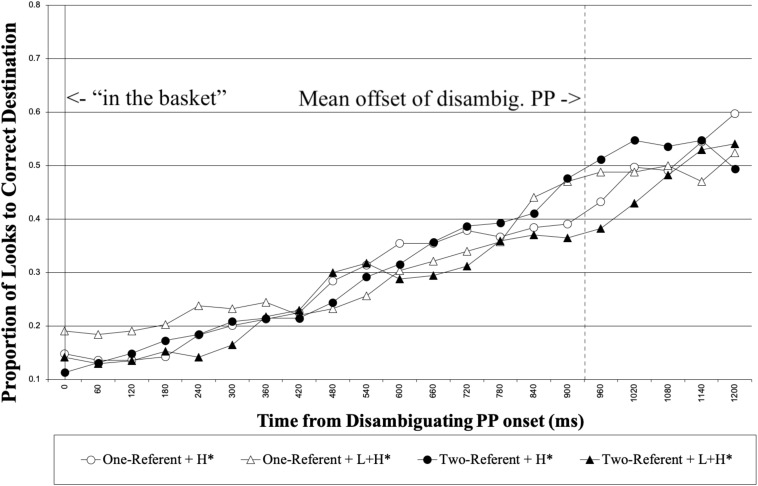
L2 learners’ proportion of looks to the correct destination object from the onset of the disambiguating PP. The right vertical line marks the mean offset of the disambiguating PP.

We next report the looks to the correct destination object for the duration of the disambiguating PP. Figure 6 shows the proportion of looks to the correct destination object for each 60 ms, time-locked to the disambiguating PP onset until 1500 ms following it. Again, we analyzed looks made to the correct destination object for the 1000 ms time interval from 200 to 1200 ms following the onset of disambiguating PP. Table 4 summarizes the results from the optimal model.

**TABLE 4:** Results from the model on looks to the correct destination object for the duration of the disambiguating PP.

	**β**	***SE***	***t***	***p***
Intercept	–0.63	0.32	–1.97	0.059
Prosody	–0.08	0.30	–0.27	0.785
Visual context	0.056	0.30	0.20	0.844
Proficiency	0.27	0.29	0.92	0.366
Prosody^∗^Visual Context	–1.43	0.59	–2.41	0.016

No main effect was observed in the analysis. The results showed that there was an interaction between Prosody and Visual Context. To explore the pattern of the interaction, we again used dummy coding for Visual Context by coding the reference level as 0. The analysis revealed that there was no simple effect of Prosody in either the one-referent or the two-referent context conditions (*p* = 0.208 in one-referent context, *p* = 0.108 in two-referent context). Thus, the significant interaction most likely suggests that the influence of prosody on the interpretation of the disambiguating PP was different between the two types of visual context. Numerically, the results show that in the two-referent context, participants tended to look less at the correct destination object with L + H^∗^ than with H^∗^. This is opposite of what was observed with the native speakers in Experiment 1.^8^

## Discussion

In the current study, we examined the influence of prosody and visually presented context information on online referential ambiguity resolution. The results from the two experiments indicated that both native speakers and L2 learners used visual context information to resolve the ambiguity of the sentence structure. This replicates the results of previous studies that showed an effect of referential context information in syntactic processing with native speakers (Tanenhaus et al., 1995) and L2 learners (Pozzan and Trueswell, 2016). Our study, however, revealed that native speakers and L2 learners showed a different pattern of results in terms of the use of prosodic information with reference to the visual context.

The results from native speakers showed that the pitch accent information was used differently depending on the context. In the context with two possible referents, the contrastive accent on the ambiguous PP highlighted the contrast between the two objects in a visual scene and, as a result, encouraged listeners to adopt the correct Modifier PP analysis. When the identical contrastive accent was used without a contrastive set in the visual scene, there was no such facilitatory effect of prosody and in fact native speakers tended to be slower to reach the correct analysis with the contrastive accent. One possible explanation for this is that native speakers tried to reconcile the incoherent use of the accent when the visual information did not provide contrastive context and as a result experienced processing difficulty. It is likely that the accent served as simple prominence in the one-referent context and emphasized the destination for the direct object NP, leading native speakers to the incorrect locative PP analysis. The results demonstrated that native speakers rapidly integrate different sources of information such as prosody, syntax, and visually presented context during comprehension. The results further showed that the same intonational cues can convey different pragmatic intentions depending on context, supporting the view that the perception of pragmatic meaning of pitch accent is closely associated to discourse context (Grice et al., 2017). The results are compatible with the hypothesis that the mapping of prosody and meaning is not simply a bottom-up processing of acoustic cues. Listeners also consider how the cues can co-occur with a specific context and adopt different pragmatic meanings accordingly.

The results from L2 learners indicated that the contrastive accent did not facilitate the processing of the ambiguous structure when it was used in the two-referent context. L2 learners adopted the incorrect Destination PP analysis when the ambiguous PP carried a contrastive accent and the context had two possible referents. This resulted in less looks to the correct destination object at the point of disambiguation. The results demonstrated that L2 learners failed to interpret the contrastive accent as signaling contrast within the visual context. Our results suggest that L2 learners were unable to coordinate prosody and visually provided contextual information and could not assign contrastive meaning to the prosodic cue. Unlike native speakers, L2 learners did not assign contrastive meaning to the L + H^∗^ accent with reference to context. We argue that the difference between native speakers and L2 learners occurred precisely in the situation where complex integration of different sources of information was required, suggesting that L2 learners have difficulty or have a deficit in their ability to coordinate different sources of information via multiple interfaces to construe the speaker’s intended meaning.

Our results are consistent with those of Pozzan and Trueswell (2016) in terms of the influence of visually provided discourse context on L2 processing. Our results extended the previous findings to a different population with a typologically different linguistic background (i.e., head-final Japanese). This thus rules out the possibility that grammatical similarities between L1 and L2 are responsible for the use of discourse information in processing syntactic ambiguities. The results provide support for incremental and interactive processing for L2 learners through which they are able to use information other than syntax in building sentence structures. The results are also inconsistent with the predictions under the SSH that L2 learners’ sentence representations are syntactically less detailed than those of native speakers. Our results suggest that as far as the processing involves the integration of visual information, L2 learners were able to build the correct syntactic structure using visually provided contrastive context.

However, L2 learners did not process the sentences in the same way as native speakers when contrastive accents needed to be integrated with reference to contextual information. One possibility for the cause of this difference is L1 transfer. Despite the differences in various prosodic features between English and Japanese, contrastive focus is realized in a highly similar fashion between the two languages, by on-focus F0 rising and post-focus reduction (e.g., Ishihara, 2003). Given the similarity in prosodic contrastive marking and previous research showing the effect of contrastive prosody on the disambiguation of an ambiguous structure (Nakamura et al., 2012), it should be relatively easy for Japanese speakers to learn the function of the contrastive accent in English (the use of linguistic elements in L2 is easy if the same elements are present in L1, known as *positive transfer*). This predicts that the L2 learners in the current study should be able to process the cue in a similar manner to native speakers. Our finding against this prediction suggests that it is unlikely that the results of the current study are due to the transfer of knowledge of Japanese prosody to the processing of English.^9^

Another possibility is that our participants had somewhat limited and insufficient language ability to correctly process the prosodic cue. Some other studies in fact reported a link between comprehenders’ performance in L2 processing and their general L2 proficiency (Reichle, 2010; Dussias et al., 2013; Reichle and Birdsong, 2014). However, our analyses showed that language proficiency level did not mediate the results of the eye-movements in any way. Although we cannot say anything conclusive considering that the proficiency level of our L2 participants was relatively homogeneous, the lack of the effect of Proficiency level might suggest that the L2 learners’ difficulty was not due to their general language proficiency.

Instead, we argue that the difference between native speakers and L2 learners is most likely due to certain processing factors; L2 learners have difficulty in processing sentences that require complex integration of multiple sources of information. The comprehension of the experimental items in the current study required readers to coordinate syntactic as well as prosodic information with visual context. Our results suggest that such highly complex integration of multiple sources of information exceeded the L2 learners’ processing capacity, which is in some sense consistent with what is predicted by the Interface Hypothesis. However, importantly, the finding of an effect of visual context on syntactic processing with L2 learners is not expected by the Interface Hypothesis because in the model, in its strict form, L2 learners are expected to be universally incapable of processing sentences whose interpretation involves the coordination of syntax with contextual information.

Our results demonstrated that L2 learners did not have difficulty at the interface between syntax and visually presented discourse context, but experienced great difficulty in integrating information through multiple interfaces (grammar-discourse context, prosody-discourse context, and grammar-prosody). We now consider two possible reasons for why L2 learners failed to integrate the contrastive pitch accent with reference to visual context. One possibility is the computation, which involves integration of syntax, discourse context, and prosody, is too costly for L2 learners and they had no cognitive resources to be allocated for processing the prosodic cues, which is possibly the least salient information. If this is the case, we might expect to find that the more proficient learners would experience less processing difficulty as their processing requires less cognitive effort. We however failed to find an effect of L2 learners’ proficiency level on their processing. Although it is difficult to completely rule out this possibility because the level of L2 participants in the current study is relatively homogeneous (the L2 participants’ mean score for the English test was 194.8 out of 200, and standard deviation was 12.4), there is no evidence to support that the results were due to the excessive processing cost imposed on the L2 learners.

Another possibility is that the results may be attributed to L2 learners’ unique processing difficulty of prosodic information. Compared to the visually presented discourse information which is highly salient and language-independent, processing of prosodic information might be highly demanding for L2 learners. Japanese speakers may use contrastive focus in processing Japanese in a similar way as English speakers, but how the contrastive prosody functions must depend on many other language-specific features such as grammar. Thus, it is not so surprising that Japanese learners could not process contrastive focus in English in the same manner as native English speakers. Given that the meaning of prosodic features is often language-specific, it is possible that prosody provides a uniquely difficult interface for L2 learners, the computation of which along with syntax or other types of non-grammatical information would cause a processing difficulty for L2 learners.

The current study showed that contrastive pitch accent used in referential context facilitated the processing of syntactically ambiguous structures for L1 speakers. However, the same information did not facilitate, and in fact disrupted, the processing for L2 learners. L2 learners adopted an incorrect syntactic analysis more often when contrastive accent was used in a contrastive context. The results suggest that L2 learners failed to integrate information in coordinating contrastive accents with the visually presented contrastive context in online syntactic processing.

## Conclusion

Our results demonstrated that a visually presented context is helpful for processing ambiguous syntactic structures for both native speakers and L2 learners. It facilitated the resolution of local syntactic ambiguity immediately at the point of ambiguity. In contrast, a contrastive pitch accent was processed appropriately with reference to referential context only by native speakers but not by L2 learners. Our results indicated that visual context influenced the interpretation of the prosodic cue for L2 learners but not in a facilitative manner; L2 learners were more likely to adopt an incorrect syntactic analysis when the prosodic cue could have helped them to adopt the correct analysis. Our results suggest that L2 learners experienced processing difficulty in the computation in which prosodic cues were needed to be integrated with syntax with reference to visual context. We conclude that while visually presented contextual information is helpful for both native speakers and L2 learners in resolving syntactically ambiguous sentence structures, the integration of prosody with reference to other sources of information is particularly difficult for L2 learners.

## Data Availability Statement

The datasets generated for this study are available on request to the corresponding author.

## Ethics Statement

This study was conducted with the approval of the Committee on the Use of Humans as Experimental Subjects of Massachusetts Institute of Technology and The University of Tokyo research subjects review board. All subjects gave consent and were compensated with monetary payment for their participation.

## Author Contributions

CN and MA conceived and planned the experiments and took the lead in writing the manuscript. CN, SF, and YH carried out the experiment. All authors provided critical feedback and helped shape the research, analysis, and contributed to the final version of the manuscript.

## Conflict of Interest

The authors declare that the research was conducted in the absence of any commercial or financial relationships that could be construed as a potential conflict of interest.

## Footnotes

1 It is also possible to interpret the structure of the sentence (4) as “put the cake on the plate that is in the basket.” However, this interpretation is disambiguated by visual context as there is no “plate that is in the basket” in the visual scene.

2 One might wonder if L + H^∗^ pitch accent could provoke a contrast between two possible destinations (“put the cake [on the napkin]” as opposed to “put the cake [in the basket]”). This is certainly possible but we think it is highly unlikely because all the items used in the current study contained different prepositions for Modifier PP and Destination PP (see Supplementary Appendix) and thus the ambiguity between the two possible destinations was resolved at the preposition before hearing the contrastive accent. In order to check this, we analyzed the looks to the correct destination object for the duration of the ambiguous PP. The results showed no effect of Prosody either with native speakers or with L2ers, confirming that L + H^∗^ accent only highlighted the contrast between the two same objects (two cakes) in the two-referent condition at least in our items.

3 We also conducted the combined analysis on the looks to the incorrect destination object for the duration the ambiguous PP (“on the plate”). The results showed a main effect of Visual Context (β = −1.33, *SE* = 0.21, *t* = −6.25, *p* < 0.001). There was also a marginal interaction between Visual Context^∗^Group (β = 0.82, *SE* = 0.43, *t* = −1.88, *p* = 0.060), suggesting that there was a tendency for native speakers to use the visual context information more than L2 learners.

4 We also conducted growth curve analysis (Mirman, 2014) and examined the looks to the incorrect destination from the onset of ambiguous PP until the 1000 ms following it. Consistent with the results of the analysis reported in the main text, the results from the growth curve analysis also showed a main effect of Visual Information (*p* < 0.001). Furthermore, the analysis also showed a significant three-way interaction between Prosody^∗^Visual Information^∗^Linear Time (*p* = 0.006). The direction of the two-way interaction between Prosody and Visual Information indicates that the difference between H^∗^ and L + H^∗^ conditions were larger in the one-referent context condition than in the two-referent context condition. Thus, this three-way interaction suggests that this interaction became more evident over time, most likely reflecting that native speakers incorrectly attached the ambiguous PP to the verb when the ambiguous PP carried the L + H^∗^ accent and visual context contained only one referent. No effect of other time terms was observed.

5 Again, we conducted growth curve analysis for the looks to the correct destination object from the disambiguating PP onset until 1300 ms following it. The results revealed a main effect of Prosody (*p* = 0.028), that of Linear Time (*p* = 0.002), and a three-way interaction between Prosody^∗^Visual Information^∗^Linear Time (*p* = 0.046). Consistent with the analysis reported in the main text, the results suggest that native speakers looked at the correct destination object more with L + H^∗^ accent than with H^∗^ accent in the two-referent condition, and they looked at the correct destination object more with H^∗^ accent than with L + H^∗^ in the one-referent condition, and this two-way interaction became more evident over time. The main effect of Linear Time indicates that native speakers looked more at the correct destination object over time. The main effect of Prosody most likely reflects the larger difference between L + H^∗^ and H^∗^ in the two-referent condition than in the one-referent condition. No effect of other time terms was observed.

6 It is also possible to interpret the results that the looks to the correct destination for the duration of the disambiguating PP simply reflects how long participants looked at the object, rather than how quickly they reached the correct analysis. If this is the case, the longer looks to the correct object would reflect processing difficulty in revising the initially adopted interpretation. In order to test this possibility, we also analyzed native speaker’s data in shorter time windows following the onset of the disambiguating PP. The analysis in the 400 ms time window following the onset of disambiguating PP, which is roughly before the participants heard the noun in the disambiguating PP (e.g., basket), showed a marginally significant interaction between Prosody^∗^Visual Context (*p* = 0.066). This interaction becomes fully significant in the 600 ms time window following the onset of the disambiguating PP (*p* = 0.036). If longer looks to the correct destination are reflecting processing difficulty, participants would look more at the basket after they hear the information about the final destination object (e.g., basket), not before. Thus, the interaction observed in the shorter time windows suggests that the proportion of looks to the correct disambiguating information following the disambiguating PP onset reflects how quickly participants reached the correct analysis.

7 For L2 learners’ data, we again conducted growth curve analysis for the same intervals as we did for native speakers’ data. The analysis for the looks to the incorrect destination object from the onset of the ambiguous PP until 1000 ms following it showed an interaction between Visual Context^∗^Linear Time (*p* = 0.045). The interaction indicates that participants looked at the incorrect destination object more in the one-referent context than in the two-referent context, and this effect became stronger over time. There was no effect of other time terms.

8 Again, we conducted growth curve analysis for the looks to the correct destination object for the same time interval as we did for the native speaker’s data. The results showed a main effect of Visual Context (*p* = 0.016) and an interaction between Prosody^∗^Visual Context (*p* = 0.033). Consistent with the analysis reported in the main text, the interaction suggests that the influence of Prosody was different between the one-referent context and two-referent conditions. There was no effect of other time terms.

9 For the L1 transfer effect, we cannot deny the possibility that the word order difference between English and Japanese might have affected the learners’ performance. In Japanese, modifying information comes before the noun whereas a modifying prepositional phrase comes after the noun in English. Because of this word order, it is possible that Japanese speakers experienced strong difficulty in processing the temporarily ambiguous PP attachment structure in English, which might have resulted in putting extra load in integrating prosody and context information.
