# Supplementary material for: An Extra Cue Is Beneficial for Native Speakers but Can Be Disruptive for Second Language Learners: Integration of Prosody and Visual Context in Syntactic Ambiguity Resolution
Source: Front Psychol. 2020 Jan 10;10:2835. doi: 10.3389/fpsyg.2019.02835 (PMC6965364; doi:10.3389/fpsyg.2019.02835)
Supplement: Supplementary file 1 [file Data_Sheet_1.docx]

**Appendix**

The 24 sets of experimental sentences used in Experiment 1 and 2. The noun within the ambiguous PP (underlined) was presented either with a new information accent or a contrastive accent. Auditory as well as visual stimuli can be obtained upon request from the first author.

1. Put the apple on the napkin in the bowl.
2. Put the wallet on the towel in the backpack.
3. Put the knife on the handkerchief in the basket.
4. Put the orange on the scarf in the pot.
5. Put the tomato on the plate in the pot.
6. Put the fork on the plate in the cup.
7. Put the candy on the hat in the trashcan.
8. Put the cake on the plate in the basket.
9. Put the spoon on the towel in the cup.
10. Put the lemon on the towel in the cup.
11. Put the ball on the chair in the bag.
12. Put the mirror on the scarf in the bag.
13. Put the camera on the hat in the backpack.
14. Put the cell phone on the envelope in the bag.
15. Put the carrot on the napkin in the pot.
16. Put the bear on the handkerchief in the box.
17. Put the chocolate on the chair in the backpack.
18. Put the pineapple on the napkin in the bowl.
19. Put the hairbrush on the envelope in the box.
20. Put the cigarette on the hat in the trashcan.
21. Put the book on the scarf in the box.
22. Put the pen on the envelope in the basket.
23. Put the strawberry on the handkerchief in the bowl.

Put the newspaper on the chair in the trashcan.
